# Proteomic and lipidomic characterization of *Hemipyrellia ligurriens* larval extracts using LC-MS/MS and GC-MS: Identification of putative antimicrobial molecules and preliminary functional assessment

**DOI:** 10.1371/journal.pone.0358738

**Published:** 2026-09-22

**Authors:** Pluemkamon Phuwanatsarunya, Sophit Khanthawong, Worasak Kaewkong, Tongjit Thanchomnang, Chaturong Inpad, Sudarat Onsurathum, Rapee Thummeepak, Ketsarin Thipphet, Kabkaew L. Sukontason, Tomomitsu Satho, Nophawan Bunchu

**Affiliations:** 1 Department of Microbiology and Parasitology, Faculty of Medical Science, Naresuan University, Phitsanulok, Thailand; 2 Department of Biochemistry, Faculty of Medical Science, Naresuan University, Phitsanulok, Thailand; 3 Faculty of Medicine and Biomedical Science Research Unit, Mahasarakham University, Maha Sarakham, Thailand; 4 Department of Parasitology, Faculty of Medicine, Chiang Mai University, Chiang Mai, Thailand; 5 Faculty of Pharmaceutical Sciences, Fukuoka University, Fukuoka, Japan; Universiti Teknologi Malaysia - Main Campus Skudai: Universiti Teknologi Malaysia, MALAYSIA

## Abstract

*Hemipyrellia ligurriens* is a tropical blow fly (Diptera: Calliphoridae) of medical and forensic importance; however, the molecular composition of its larvae remains poorly characterized. Here, we provide the first integrative proteomic and lipidomic dataset describing the molecular composition of *H. ligurriens* larvae and comparing whole-body extracts (WE) with excretory-secretory (ES) products. Label-free LC-MS/MS analysis identified 9,444 proteins in WE and 10,977 proteins in ES products, revealing 7,896 shared proteins together with 1,548 WE-specific and 3,081 ES-specific proteins. The proportion of ES-specific proteins was significantly higher than that of WE-specific proteins (*P* < 0.001), highlighting pronounced compartment-specific molecular diversity. Among proteins with molecular weights below 20 kDa, several putative antimicrobial peptides and immune-associated proteins were detected, including defensin-like, diptericin-like, and attacin-like sequences, as well as lysozyme isoforms, serine proteases, and oxidative stress-related enzymes. Lipidomic profiling of WE by GC-MS identified 26 fatty acids, comprising both saturated and unsaturated fatty acids. Functional assays demonstrated selective but moderate antibacterial activity: WE inhibited *Bacillus subtilis* and *Pseudomonas aeruginosa*, whereas ES products inhibited only *B. subtilis* (MIC > 400 μg/ml), and no antifungal activity was detected under the tested conditions. Cytocompatibility assays in HaCaT keratinocytes showed no cytotoxic effects of WE across tested concentrations and increased MTT-based metabolic activity after 24 h exposure, whereas ES products slightly reduced cell viability at the highest concentration tested (100 μg/ml). These findings support compartment-specific molecular organization between larval tissues and secretions, expand the molecular resources available for tropical calliphorid flies, and provide a foundation for future purification, functional validation, and biomedical investigations.

## Introduction

Blow flies (Diptera: Calliphoridae) develop in microbe-rich environments such as carrion, decomposing organic matter, and animal wounds [1]. During larval development, these insects are continuously exposed to diverse microbial communities and must rely on efficient innate immune mechanisms to survive in microbe-dense substrates [2,3]. As a consequence, blow fly larvae produce a wide range of defense-associated molecules that regulate microbial growth while maintaining compatibility with host tissues, an attribute that has attracted increasing interest for biomedical applications. Dipteran larvae are known to produce antimicrobial peptides (AMPs), lysozymes, proteases, and oxidative stress-related enzymes that contribute to microbial regulation and environmental adaptation [4–7]. Beyond their antimicrobial activity, larval extracts have been reported to exhibit antioxidant effects [8**]**, attributed to the presence of free-radical-scavenging enzymes and small-molecule antioxidants, which may reduce oxidative stress, a key factor contributing to impaired wound healing and chronic inflammation. In addition, larval secretions have been reported to promote fibroblast proliferation, angiogenesis, and extracellular matrix remodeling, thereby facilitating tissue repair and regeneration [9]. Among these molecules, antimicrobial peptides represent a central component of dipteran innate immunity [7]. These small, typically cationic peptides, often below 20 kDa, act by disrupting microbial membranes or interfering with essential cellular processes [10]. In blow flies, defensins (e.g., lucifensin), diptericin, cecropins, and attacin have been identified as key mediators of antibacterial activity against both Gram-positive and Gram-negative bacteria [11–13]. Because these molecules are frequently enriched in excretory-secretory (ES) products and may also occur systemically within larval tissues [14, 15], comprehensive analysis of both secreted and whole-body fractions is required to fully characterize the larval antimicrobial repertoire.

Molecular studies in calliphorid flies have largely focused on a limited number of species, particularly *Lucilia sericata* and *Lucilia cuprina*, in which antimicrobial peptides and various larval-derived biomolecules have been partially characterized [5,11,14,16]. More recent studies have further investigated the antimicrobial activities of synthetic peptides derived from these flies and their potential therapeutic applications [17,18]. The larvae of *Chrysomya albiceps* secrete a diverse repertoire of bioactive molecules, including AMPs, proteolytic enzymes, and immune-modulating compounds. These secretions have demonstrated broad-spectrum antibacterial activity against Gram-positive and Gram-negative bacteria, including drug-resistant strains [8,19,20]. Recent studies have demonstrated that Larveel**®**, a GMP-produced lyophilized extract derived from *L. sericata* larvae, promotes wound debridement, granulation, and wound healing, with its therapeutic effects being associated with antibiofilm activity, highlighting the biomedical potential of blow-fly-derived biomolecules **[**21**]**. In contrast, many tropical blow fly species of medical and forensic importance remain poorly investigated at the molecular level. One such species is *Hemipyrellia ligurriens*, a widespread Old World blow fly commonly associated with decomposing substrates and forensic investigations [22–24]. Given its phylogenetic proximity to *L. sericata* and its ecological adaptation to microbe-rich environments [25], *H. ligurriens* larvae are expected to produce a diverse repertoire of antimicrobial and defense-related molecules. Although its morphology, development, and microbiome have been previously described [24,25], comprehensive proteomic and lipidomic characterization of its larval biochemical composition remains lacking.

Larval bioactive molecules are distributed across distinct biological compartments. Excretory-secretory products represent actively released proteins and peptides that interact with the external environment [26,27], whereas whole-body extracts (WE) contain intracellular and tissue-associated molecules derived from the fat body, digestive tract, salivary glands, haemolymph, and other organs [5,11,14,15,28,29]. Comparative profiling of these fractions can therefore provide insights into compartment-specific molecular allocation. In addition to proteins and peptides, lipids represent another important class of bioactive molecules in insects [30]. Beyond their structural and metabolic roles, fatty acids and related lipid components may contribute to antimicrobial defense, membrane regulation, signaling, and interactions with microbes in the larval microenvironment [31,32**]**. Several saturated and unsaturated fatty acids have been reported to exhibit antibacterial activity in diverse biological systems, including insect-derived extracts [30,33]. However, compared with protein- and peptide-based studies, lipidomic information on calliphorid larvae remains limited. Characterizing larval lipid composition may therefore provide complementary insight into the molecular basis of antimicrobial activity and broaden understanding of the bioactive repertoire of medically important blow flies.

Advances in high-resolution mass spectrometry now enable large-scale molecular characterization of non-model organisms through homology-based database matching, even in the absence of fully annotated genomes. Integrating proteomic and lipidomic analyses with functional assays provides a powerful approach for exploring larval biochemical composition and biological activity. We hypothesized that WE and ES products harbor distinct classes of bioactive molecules that contribute differently to antimicrobial activity. To test this hypothesis, we integrated proteomic, lipidomic, antimicrobial, and cytocompatibility analyses. In the present study, we performed comparative proteomic profiling of WE and ES products of third-instar *H. ligurriens* larvae using label-free LC-MS/MS. Lipidomic analysis was then conducted in WE using GC-MS. Antimicrobial and cytocompatibility assays were performed to evaluate biological activity in WE and ES products. This study provides the first integrative proteomic and lipidomic dataset for *H. ligurriens* larvae and offers new molecular insights into antimicrobial and wound-associated bioactive molecules from a medically and forensically important yet understudied tropical blow fly.

## Materials and methods

### Rearing of flies in the laboratory

The colony of *H. ligurriens* was maintained under laboratory conditions as previously described [24] at the Department of Microbiology and Parasitology, Faculty of Medical Science, Naresuan University, Phitsanulok, Thailand. All adult flies were initially identified morphologically using a taxonomic key [34], and species identity was subsequently confirmed by DNA barcoding, the results of which were reported previously [25]. Adults were provided with a 10% (w/v) sugar solution supplemented with 5% (v/v) multivitamin syrup (SEVEN SEAS, Thailand) and fed *ad libitum* with fresh pork liver (SK Interfood, Thailand). Larvae were reared on antibiotic-free fresh pork liver under controlled conditions at 24–28 ± 0.5°C, 60–70% relative humidity, and a 12-h light/dark cycle. Only third-instar (3–5 days old) were used for all experiments. All experimental procedures were approved by the Naresuan University Animal Care and Use Committee (NUACUC; Protocol No. NU-AI650911).

### Preparation of larval extracts and protein determination

The WE and ES products from third instars (3–5 days old) of *H. ligurriens* were prepared following the method described by [35], with minor modifications. Absolute methanol was selected as the extraction solvent based on previous studies demonstrating its effectiveness in recovering antimicrobial bioactive compounds from fly larvae [36]. For WE preparation, 400 inactivated third instars (approximately 10 g) were homogenized in absolute methanol (RCI Labscan, Thailand) (2:1, v/w) and incubated overnight at room temperature under orbital shaking. The homogenate was centrifuged using a High Speed Refrigerated Micro Centrifuge at 4,000 × *g* for 30 min at 4°C (TOMY MX-301, Japan). The supernatant was collected and concentrated using a rotary evaporator (Hei-VAP Core, Heidolph, Korea), followed by further solvent removal using a centrifugal evaporator (CVE-2200, Eyela, Japan). The dried extract was resuspended in 5% dimethyl sulfoxide (DMSO) (RCI Labscan, Thailand) and sterile-filtered prior to use. For ES products collection, third instars were surface-sterilized with 70% ethanol (RCI Labscan, Thailand) and rinsed three times with sterile distilled water. Larvae were transferred into sterile 50 ml tubes (100 larvae in 100 µl sterile water per tube), with a total of 2,000 larvae processed. Samples were incubated for 60 min at 24–28°C in the dark to allow secretion. The pooled ES products were supplemented with a protease inhibitor cocktail (AMRESCO, OH, USA) and centrifuged at 1,300 × *g* for 5 min at 4°C. The supernatant was filtered through a 0.45 µm syringe filter (Sartorius, Germany) and tested for sterility by plating 100 µL on blood agar and brain-heart infusion agar (Himedia, India), followed by incubation at 37°C for 48 h prior to antimicrobial assays.

Protein concentrations were determined using the Bradford assay (Bio-Rad, CA, USA) with bovine serum albumin (BSA; Sigma-Aldrich, USA) as the standard. Absorbance was measured at 595 nm, using SpectraMax^®^ ABS and ABS Plus absorbance microplate readers (Molecular Devices, CA, USA) and protein concentrations were calculated based on a BSA standard curve. Both WE and ES fractions were subjected to proteomic profiling, antimicrobial assays, and cytocompatibility evaluation. Lipidomic analysis was performed exclusively on the WE fraction.

### Mass spectrometry label-free protein quantification

In-solution digestion was performed using 50 µg of protein per sample according to a previously described method [35]. Peptides were analyzed using a nano-liquid chromatography-tandem mass spectrometry (LC-MS/MS) system, which consisted of a Dionex Ultimate 3000 RSLCnano System (Thermo Scientific) and a CaptiveSpray source/Quadrupole ion trap mass spectrometer (Q-ToF Compact, Bruker, Germany). One µg of peptides was enriched on a Nano trap column and separated on a PepMap100 C18 LC column. Elution was performed with a 2–95% Solvent B gradient over 160 min at a flow rate of 300 nl/min and at 60°C. The mobile phases used were A) 0.1% formic acid in water and B) 0.08% formic acid in 80% acetonitrile. The gradient for mobile phase B was as follows: 2% (5 min), 30% (130 min), 50% (10 min), 70% (5 min), 95% (5 min), followed by a rapid decrease to 2% and re-equilibration (5 min). The drying gas was set to 5 l/min at 150°C, with a nebulizer gas pressure of 0.2 bars. MS acquisition was carried out in positive ionization mode at 6 Hz, with a mass range of m/z 150–2200. AutoMSn CID fragmentation was performed at low (4 Hz) and high (16 Hz) rates for the top two precursor ions, with a three-second dynamic exclusion. Raw data were processed using MaxQuant (version 1.6.2.10) with the Andromeda search engine against the UniProt Calliphoridae database, acknowledging the presence of uncharacterized proteins typical of non-model organisms. The default setting, with the Calliphoridae database downloaded from www.uniprot.org, was set to 1 as a label-free approach. Parameter settings used the defaults except the following. Parameter settings used the defaults except the following. Search parameters included carbamidomethylation of cysteine as a fixed modification, and methionine oxidation and N-terminal acetylation as variable modifications. The instrument type was set to Bruker Q-TOF, with peptide mass tolerances of 0.5 for the first search and 0.25 for the main search. Trypsin/P was specified with up to two missed cleavages. The false discovery rate (FDR) was set at 1% at the protein level. TOF MS/MS match tolerance was set at 40 ppm with label-free quantification. A match between run options in the software was used to recalibrate mass and retention times between runs. Protein identification was performed through homology-based database matching. Therefore, assigned identities represent putative annotations, and species matches correspond to available Calliphoridae database entries rather than confirmed species origin. Proteins with molecular weights below 20 kDa were further analyzed due to their relevance to antimicrobial and immune-related functions, and database annotation identified members of the attacin, diptericin, and defensin families [6]. Proteomic analysis was performed on one pooled sample per fraction, with each sample analyzed in three technical replicates by LC-MS/MS.

### Component analysis of the fatty acid extracts by GC-MS

Lipidomic analysis was performed exclusively on the WE fraction because whole-body extracts derived from multiple larval tissues are likely to contain a broader range of lipid-associated molecules than ES products. Previous studies have similarly characterized larval lipids using whole-body or tissue-derived extracts [30,37,38], and methanol extraction has been widely used for fatty acid characterization in insects [39]. Accordingly, we focused on methanol-derived WE for fatty acid characterization. Fatty acid analysis of the WE was performed using an in-house method based on AOAC 996.06 [40]. Methanol-derived WE prepared as described above was subjected to fatty acid methylation prior to GC analysis. For sample preparation and drying, the extracted fat residue was dissolved in 2–3 ml of chloroform and 2–3 ml of diethyl ether, transferred to a 3-dram glass vial, and evaporated to dryness in a 40 °C water bath under nitrogen. For fatty acid methylation, the dried residue was derivatized to fatty acid methyl esters (FAMEs) by adding 2.0 ml of 7% boron trifluoride (BF_3_) reagent and 1.0 ml of toluene. The vial was sealed with a screw-cap containing a Teflon/silicone septum and heated at 100 °C for 45 min, with gentle shaking every 10 min. After methylation, the vial was cooled to room temperature (20–25 °C), and 5.0 ml of distilled water, 1.0 ml of hexane, and 1.0 g of sodium sulfate were added. The mixture was shaken for 1 min to facilitate phase separation. The upper hexane layer containing FAMEs was carefully transferred to another vial containing 1.0 g of sodium sulfate and prepared for GC analysis. For GC analysis, a 2 µl aliquot of both standard and test sample solutions was injected into the GC system, and mixed FAME standards were used to optimize chromatographic conditions prior to sample analysis. The methyl esters were quantified using a gas chromatograph fitted with an SP2560 fused capillary column (100 m × 0.25 mm, 0.20 μm film). The carrier gas, helium, was set at a flow rate of 1.1 ml/min with a split ratio of 100:1. The initial column temperature was maintained at 140 °C for 5 min; then, it was increased at 4 °C/min to 240 °C and held for 20 min. Fatty acids were identified by matching retention times with commercial FAME standards and reported as relative percentages. The limit of detection was 0.01 g per 100 g of extract.

### Antimicrobial susceptibility test

#### Microbial cultures and strains used in the study.

This study employed a variety of microorganisms, including bacteria, yeast, and filamentous fungi, which were provided by the Department of Microbiology and Parasitology, Faculty of Medical Science, Naresuan University, Thailand. Representative Gram-positive, Gram-negative, yeast, and filamentous fungal strains were selected to assess antibacterial and antifungal activity. The bacterial strains included *Bacillus subtilis* DMST 5871, *Staphylococcus epidermidis* DMST 15505, *Staphylococcus aureus* TISTR No. 1466, *Pseudomonas aeruginosa* TISTR No. 1467, *Escherichia coli* TISTR No. 887, and *Proteus vulgaris* DMST 5685. The yeast strains included *Candida albicans* DMST 8684, while the filamentous fungi included *Aspergillus flavus* DMST 22950 and *Penicillium* sp. Bacterial cultures were grown on nutrient agar (Himedia, India). All bacterial cultures were incubated at 37°C for 24 h in an incubator (Model 1915, Sheldon Manufacturing Inc., USA) before use. Yeast strains were cultured on Sabouraud Dextrose Agar (SDA) (Himedia, India) at 37°C for 24 h, while filamentous fungi were grown on SDA and incubated at room temperature for 48–72 h. The study was approved by the Naresuan University Institutional Biosafety Committee (NUIBC, Protocol No. MI 66-12-55) to ensure adherence to biosafety standards.

### Agar well diffusion (AWD) assay

The agar well diffusion assay was performed using a modified method based on Teh et al. [41]. Bacterial suspensions were prepared at a McFarland standard 0.5 (10^8^ cells/ml) using a DEN-1B McFarland densitometer (BioSan, Riga, Latvia), while yeast suspensions were prepared at a McFarland standard of 1 (10^6^ cells/ml). The standardized suspensions were evenly spread onto Mueller-Hinton agar plates (Himedia, India). Wells of 4 mm in diameter were punched into the agar, and a fixed application volume of 100 µl per well of either WE or ES products was added to each well (198 µg WE or 175 µg ES products per well). Tetracycline (30 μg) (Oxoid, UK) and ciprofloxacin (5 μg) discs (Himedia, India) were used as positive controls for antibacterial activity, and fluconazole (25 μg) discs (Himedia, India) were used as antifungal controls. Sterile 5% DMSO and distilled water served as negative controls for WE and ES products, respectively. Plates were incubated at 37°C for 24 h (bacteria) or 30°C for 48 h (yeasts). Inhibition zones were measured using a digital vernier caliper (OKURA, Japan). For filamentous fungi, a 6-mm mycelial plug, cut with a 6-mm cork borer, was placed at the center of a SDA plate and incubated at room temperature for two days to allow initial growth. Subsequently, 4-mm wells were punched into the agar, and 100 µl of larval extracts was added to each well as described above. SDA supplemented with cycloheximide (0.5 mg) (Himedia, India) was used as the positive control. Plates were incubated at room temperature for 2–6 days. Fungal growth inhibition was visually assessed and compared with control plates. All assays were performed in triplicate. Only microorganisms showing susceptibility to the larval extracts, as indicated by the presence of inhibition zones or absence of visible growth, were selected for determination of the minimum inhibitory concentration (MIC) and minimum bactericidal concentration (MBC).

### Broth dilution method for MIC and MBC determination

Based on the results of the agar well-diffusion assay, only *B. subtilis* and *P. aeruginosa* were selected for MIC and MBC determination using the resazurin-based turbidimetric (TB) assay. The assay was adapted from the method described by Teh et al. [42]. Broth microdilution was performed in accordance with the Clinical and Laboratory Standards Institute (CLSI) guidelines. The extracts were serially diluted in Mueller-Hinton broth (MHB) to obtain final concentrations ranging from 50 to 400 μg/ml. A standardized bacterial suspension (1 × 10⁶ cells/ml) was added to all test tubes, except for the broth sterility control and extract sterility control. Growth controls containing bacterial suspension in MHB without sample extracts were included for each bacterial strain. Following overnight incubation at 37°C, resazurin solution (6.75 mg/ml) (Sigma-Aldrich, Steinheim, Germany) was added to each tube, and incubation was continued for an additional 4 h at 37°C. Color changes of resazurin were visually assessed and recorded. The MIC was defined as the lowest extract concentration at which no color change of resazurin (blue to pink) was observed relative to the control. To determine the MBC, 1 μl from each tube showing no visible growth was inoculated onto nutrient agar plates using a calibrated loop. After 24 h incubation at 37°C, the plates were examined for bacterial growth. Due to limited sample availability, MIC and MBC determinations were performed in duplicate.

### Cytotoxicity analysis by MTT assay

The cytotoxicity of WE and ES products was evaluated using HaCaT cells (immortalized human keratinocytes; BIOTEC, Thailand), a widely used *in vitro* model for assessing cytocompatibility in skin- and wound-related studies [43]. Cytotoxicity was evaluated using an MTT (3-[4,5-dimethylthiazole]-2,5-diphenyltetrazolium bromide) assay, as previously described by Phuwanatsarunya et al. [35]. Cells were seeded in 96-well plates at a density of 2 × 10^3^ cells per well and allowed to attach for 24 h at 37 °C in a 5% CO_2_. Then, the culture medium was removed, and the cells were treated with various concentrations of WE and ES products (0.1–100 µg/ml) for 24 h. This concentration range was selected based on a previous MTT study [35] and used for preliminary cytocompatibility screening of the larval extracts. Cells were then administered 10 µl of 0.5 µg/ml MTT reagent (Bio Basic, Markham, Canada) and left for 3 h in the dark at 37°C. Media were then carefully discarded, and DMSO (Sigma-Aldrich, MO, USA) was added to each well to dissolve the formazan crystals. Absorbance was measured at 540 nm. Data are presented as mean ± standard deviation from biological triplicates.

### Statistical analysis

Unless otherwise stated, all proteomic data processing, statistical analyses, and figure generation were performed in Python version 3.9 using NumPy, pandas, SciPy, and Matplotlib. Statistical significance was assessed at α = 0.05, and all reported *P* values were two-tailed. The Python source code and accompanying input data used for statistical analyses and data visualization in this study are provided in S1 Code.

Label-free LC-MS/MS intensities obtained from the MaxQuant proteinGroups table were log-transformed. Relative protein abundance between WE and ES products was expressed as log_2_(ES/WE) fold change following addition of a uniform pseudocount. Proteins with |log_2_ fold change| ≥ 1 and a combined intensity ≥ 1 × 10^6^ were classified as abundance-enriched candidates for exploratory visualization. The statistical significance of the overlap between WE and ES proteomes was assessed using a hypergeometric test with the union of all detected proteins as the background population. Differences in the proportions of compartment-exclusive proteins were evaluated using a two-proportion z-test, and overlap similarity was quantified using the Jaccard coefficient.

Antibacterial activity in the agar-well diffusion assay was expressed as mean inhibition-zone diameter ± standard deviation from three independent experiments.

For the HaCaT keratinocyte MTT assay, cell viability at each extract concentration was expressed as a percentage and presented as mean ± standard deviation from three independent biological replicates. Statistical analyses were performed using GraphPad Prism (version 8), with Microsoft Excel (version 16.6.1, 22101101) used for data organization. Statistical significance was assessed using Student’s *t*-test for independent pairwise comparisons between each treatment concentration and the untreated control group. *p* < 0.05 was considered to indicate a statistically significant difference. MTT assay figures were generated in Python version 3.9.

## Results

### Yield and properties of larval extracts

Whole-body extraction of *H. ligurriens* larvae yielded 780 ± 2.99 mg of extract, corresponding to 7.8% of the initial larval material (400 larvae). The average volume of ES products obtained from 2,000 larvae was 5.97 ± 1.10 ml, corresponding to an estimated yield of approximately 2.99 µl per larva. In terms of protein, the total protein content from the WE was 2.29 (1.84–7.10) mg/ml. The total protein content of ES products was 2.72 (1.75–6.22) mg/ml or 8.12 µg per larva.

### Protein profiles of larval extracts

**Fig 1:**
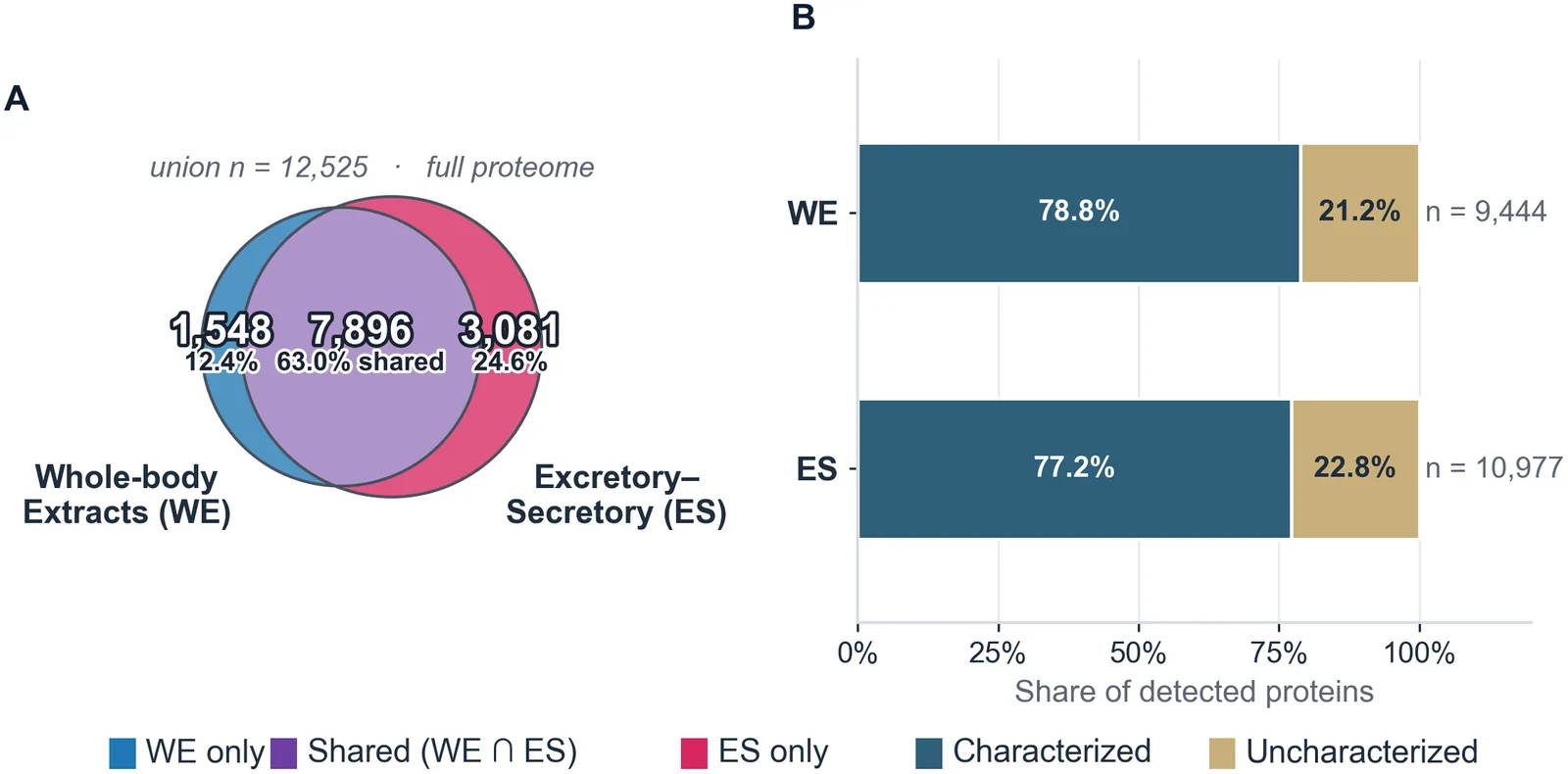
Total-proteome characterization of *H. ligurriens* larval WE and ES product fractions. (A) Venn diagram illustrating shared and uniquely identified proteins and their compartmental partitioning between WE and ES fractions (union n = 12,525). (B) Percentage distribution of characterized and uncharacterized proteins per fraction based on LC-MS/MS analysis and UniProt Calliphoridae database annotation.

Proteomic profiling of WE and ES products from *H. ligurriens* larvae using LC-MS/MS revealed a high diversity of proteins. Venn diagram analysis showed that 7,896 proteins (63.0%) were shared between WE and ES samples. In contrast, 1,548 proteins (12.4%) were uniquely identified in WE, whereas 3,081 proteins (24.6%) were detected exclusively in ES products (Fig 1A). A total of 9,444 proteins were identified in WE, including 7,440 characterized proteins (78.8%) and 2,004 uncharacterized proteins (21.2%). In ES products, 10,977 proteins were detected, of which 8,474 (77.2%) were characterized and 2,503 (22.8%) were uncharacterized (Fig 1B). To evaluate compartment-specific protein distribution, the proportion of fraction-exclusive proteins was compared between ES and WE products. ES products contained a significantly higher proportion of exclusive proteins (28.1%, 3,081/10,977) than WE products (16.4%, 1,548/9,444) (two-proportion z-test, *P* < 0.001). The Jaccard overlap coefficient was 0.630, indicating substantial overlap between fractions despite asymmetry in protein exclusivity and suggesting compartment-specific protein distribution.

**Fig 2:**
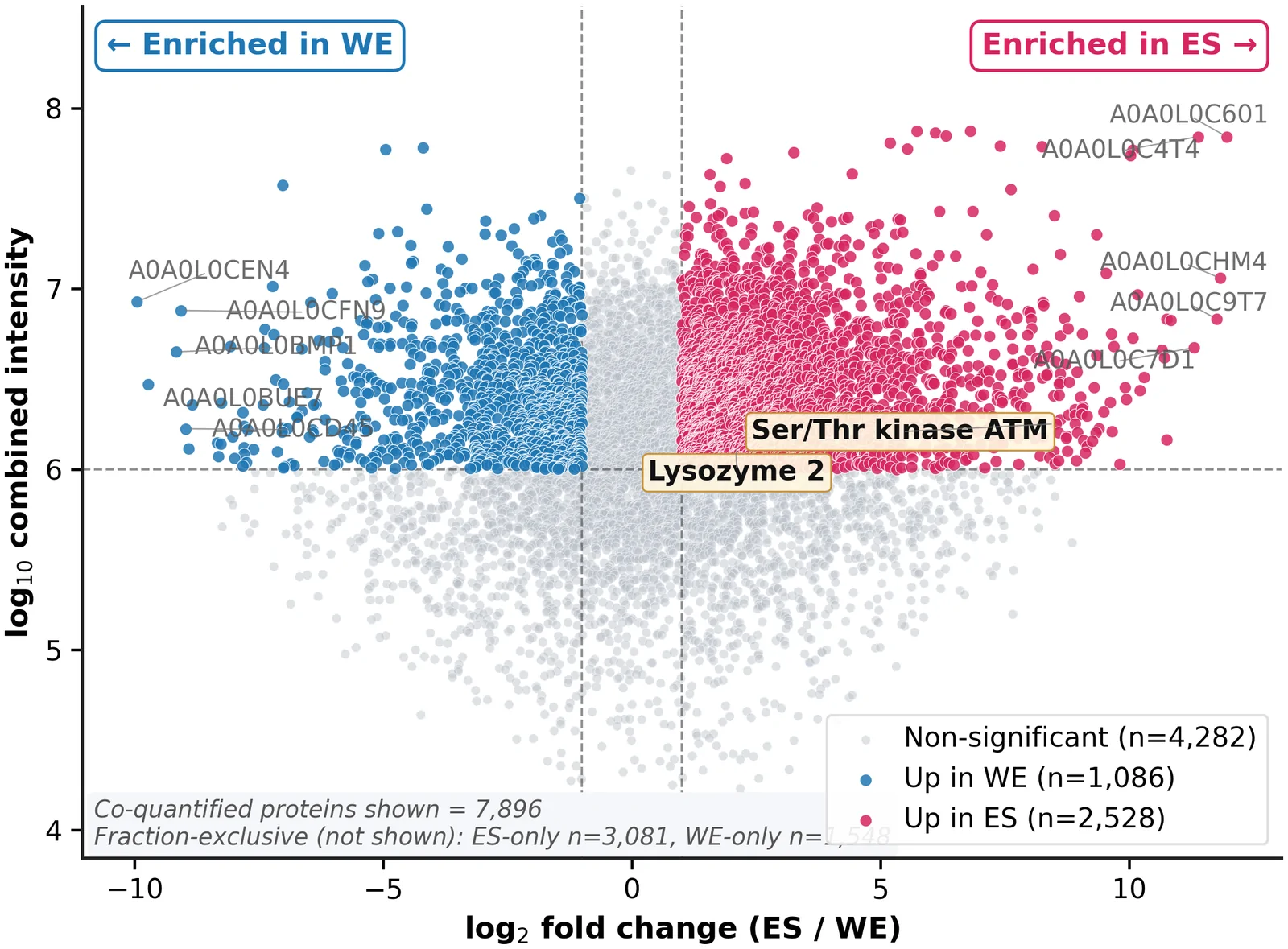
Differential abundance profiling of co-quantified proteins between WE and ES products of *H. ligurriens* larvae. Volcano-style exploratory plot of 7,896 co-quantified proteins. Each point represents one protein. The x-axis shows log_2_-transformed fold change (ES/ WE intensity), and the y-axis shows log_10_ combined LC-MS signal intensity. Threshold lines indicate fold-change (|log_2_ FC| = 1) and absolute-intensity (≥ 10^6^) cut-offs. Proteins enriched in ES (positive log_2_ FC; magenta) and WE (negative log_2_ FC; blue) are shown, with the top five most differentially abundant proteins by absolute log_2_ FC labeled as grey UniProt accessions. Putative antimicrobial, immune-effector, and proteolytic-enzyme candidates from Table 1 within the differential-abundance region are highlighted in gold-bordered boxes and labeled by curated family or protein names (e.g., Lysozyme 2 and Ser/Thr kinase ATM).

Volcano-style analysis of the 7,896 co-quantified proteins revealed marked differences in relative protein abundance between WE and ES fractions (Fig 2). Among these, 2,528 proteins exhibited ≥2-fold higher abundance in ES products with combined intensities ≥10^6^, whereas 1,086 proteins showed ≥2-fold enrichment in WE. The remaining 4,282 co-quantified proteins fell within the |log_2_FC| < 1 or intensity <10^6^ regions and therefore displayed relatively comparable abundance between fractions. Proteins with positive log_2_ fold-change values were enriched in ES, whereas those with negative values showed greater abundance in WE. The five proteins with the largest absolute log_2_ fold-change values on each side of the distribution represented the most strongly enriched proteins in the respective fractions. Several putative antimicrobial, immune-effector, and proteolytic-enzyme candidates identified in Table 1 were located within the enrichment regions, including Lysozyme 2 and serine/threonine-protein kinase ATM, highlighting potential functional differences between WE and ES products.

**Table 1:** Putatively identified antimicrobial peptides/enzymes and growth factor- or signaling-associated proteins detected in WE and ES products of *H. ligurriens* larvae by LC-MS/MS analysis (MaxQuant with the Andromeda search engine against the UniProt Calliphoridae database).

Category 1: Antimicrobial peptides/ enzymes							
No. selected sequence	Molecular weight (kDa)^a^	Accession number^b^	Putative protein name^b^	Family	Species match^b^	Intensity of larval extracts^a^	
						WE	ES
1.	4.47	C0HJX9	Diptericin	Diptericin	*Calliphora vicina*	2.63 × 10^5^	n.d.
2.	10.73	P18684	Diptericin-D	Diptericin	*Protophormia terraenovae*	n.d.	5.21 × 10^3^
3.	10.23	A0A7D5FFX3;P86471;A0A0L0BSV1;A0A0L0CI08	Lucifensin	Defensin	*Lucilia sericata*	1.36 × 10^4^	n.d.
4.	10.11	P10891	Phormicin	Defensin	*Protophormia terraenovae*	1.33 × 10^5^	n.d.
5.	10.41	A0A0L0C905	Defensins family	Defensin	*Lucilia cuprina*	n.d.	4.22 × 10^5^
6.	13.16	A0A0L0BRJ2	Defensins family	Defensin	*Lucilia cuprina*	n.d.	8.10 × 10^5^
7.	13.97	D9J150;D9J145	Ferritin	Ferritin	*Lucilia sericata*	2.12 × 10^5^	n.d.
8.	15.63	A0A0L0BQ22	Superoxide dismutase (Cu-Zn)	SOD	*Lucilia cuprina*	1.47 × 10^5^	2.33 × 10^3^
9.	7.90	A0A0L0BNM1	Lysozyme	Lysozyme	*Lucilia cuprina*	n.d.	1.73 × 10^5^
10.	15.96	C0HLB7	Lysozyme 2	Lysozyme	*Lucilia sericata*	2.55 × 10^5^	1.09 × 10⁶
11.	11.29	D9J148	Putative lysozyme 2	Lysozyme	*Lucilia sericata*	n.d.	6.64 × 10^5^
12.	16.14	D9J143	Lysozyme 1B	Lysozyme	*Lucilia sericata*	9.89 × 10^4^	1.15 × 10^5^
13.	16.23	A0A0L0CIT1	Lysozyme D	Lysozyme	*Lucilia cuprina*	7.96 × 10^4^	9.65 × 10^4^
14.	18.17	A0A0L0C6T8	Attacin-A	Attacin	*Lucilia cuprina*	n.d.	1.14 × 10⁶
15.	20.66	A0A0L0BXA2	Attacin C	Attacin	*Lucilia cuprina*	2.57 × 10^5^	1.02 × 10^5^
16.	13.59	A0A0L0C799	Attacin C	Attacin	*Lucilia cuprina*	n.d.	2.19 × 10^4^
17.	13.71	A0A0L0C779	Attacin C	Attacin	*Lucilia cuprina*	n.d.	2.36 × 10^5^
18.	3.81	A0A0L0BYH9	Metallothionein-1	Metallothionein	*Lucilia cuprina*	n.d.	4.71 × 10^4^
19.	5.04	A0A0L0BYA8	Metallothionein-1	Metallothionein	*Lucilia cuprina*	4.47 × 10^3^	2.20 × 10^4^
20.	4.57	A0A0U3AZ32	Metallothionein-like protein	Metallothionein	*Chrysomya megacephala*	4.18 × 10^3^	1.18 × 10^5^
21.	15.21	Q25237;Q25228	Serine proteinase	Protease	*Lucilia cuprina*	n.d.	1.94 × 10^4^
22.	16.17	A0A0L0BYT7	Non-specific serine/threonine protein kinase	Protein kinase	*Lucilia cuprina*	n.d.	9.05 × 10^4^
23.	8.06	A0A7D5FH37	Chymotrypsin	Protease	*Lucilia sericata*	n.d.	1.30 × 10^4^
24.	18.20	Q9GSL6;Q9GSM2	Serine protease K16/F1R2	Protease	*Chrysomya bezziana*	n.d.	7.14 × 10^4^
25.	18.71	Q9GSN2	Serine protease K13/F2R3	Protease	*Chrysomya bezziana*	n.d.	2.34 × 10^5^
26.	19.11	Q25231	Serine proteinase	Protease	*Lucilia cuprina*	1.76 × 10^5^	5.68 × 10^4^
27.	19.37	Q9GSM7;Q9GSM0	Serine protease K16/F1R1	Protease	*Chrysomya bezziana*	n.d.	1.49 × 10^4^
28.	19.60	Q9GSM9	Serine protease K15/F2R3	Protease	*Chrysomya bezziana*	n.d.	1.03 × 10^5^
29.	19.75	Q9GSM8	Serine protease K3/F1R1	Protease	*Chrysomya bezziana*	n.d.	1.03 × 10^6^
30.	19.84	Q9GSL7;Q9GSL8	Serine protease K12/F2R1	Protease	*Chrysomya bezziana*	3.21 × 10^5^	n.d.
31.	19.90	Q9GSL9	Serine protease K14/F2R1	Protease	*Chrysomya bezziana*	n.d.	1.80 × 10^5^
32.	20.04	A0A0L0C1F0	Serine/threonine-protein kinase ATM	Protein kinase	*Lucilia cuprina*	4.75 × 10^3^	1.78 × 10^6^
33.	13.72	A0A0L0BQK7	Insulin-like domain-containing protein	Insulin-like	*Lucilia cuprina*	n.d.	6.58 × 10^3^
34.	18.13	A0A0L0C4Y9	Putative insulin-like peptide 7	Insulin-like	*Lucilia cuprina*	3.34 × 10^5^	n.d.
**Category 2: Growth factor-related/ signaling-associated proteins**							
**No. selected sequence**	**Molecular weight (kDa)** ^**a**^	**Accession number** ^**b**^	**Putative protein name** ^**b**^	**Family/ group**	**Species match** ^**b**^	**Intensity of larval extracts** ^**a**^	
						**WE**	**ES**
1.	330.27	A0A0L0C4T6	Multiple epidermal growth factor-like domains protein 8	EGF-related	*Lucilia cuprina*	9.76 × 10^4^	3.28 × 10^6^
2.	43.61	A0A0L0C8Y3	Acidic fibroblast growth factor intracellular-binding protein	FGF-binding	*Lucilia cuprina*	4.20 × 10^5^	9.61 × 10^5^
3.	47.26	A0A0L0C6G4	Fibroblast growth factor	FGF	*Lucilia cuprina*	1.89 × 10^5^	2.76 × 10^5^
4.	84.29	A0A0L0BYZ0	Fibroblast growth factor receptor	FGF	*Lucilia cuprina*	1.35 × 10^6^	1.22 × 10^6^
5.	100.16	A0A0L0BZY8	Transforming growth factor-beta-induced protein ig-h3	TGF-β	*Lucilia cuprina*	2.96 × 10^5^	3.25 × 10^6^
6.	37.63	A0A0L0CFS2	Transforming growth factor beta regulator 1	TGF-β	*Lucilia cuprina*	n.d.	4.16 × 10^4^
7.	103.52	A0A0L0C9B0	Platelet-derived growth factor (PDGF) family profile domain-containing protein	PDGF	*Lucilia cuprina*	8.80 × 10^4^	3.51 × 10^5^
8.	202.15	A0A0L0BVB5	Vascular endothelial growth factor receptor 1	VEGF	*Lucilia cuprina*	2.92 × 10^6^	3.63 × 10^5^
9.	23.44	A0A0L0BWJ2	Gamma-interferon-inducible lysosomal thiol reductase	IFN-γ-related	*Lucilia cuprina*	2.77 × 10^4^	3.37 × 10^5^
10.	61.22	A0A0L0CPT6	Insulin-like growth factor-binding protein complex acid labile subunit	IGF-binding	*Lucilia cuprina*	n.d.	3.22 × 10^5^

**WE**: whole body extracts; **ES**: excretory-secretory product

a Proteins identified by LC-MS/MS in the present study.

b Closest annotated homolog in the UniProt Calliphoridae database. Protein identities are putative and based on sequence homology; species matches reflect database availability, not confirmed origin.

n.d. (intensity = 0) indicates non-detection in the corresponding fraction.

**Fig 3:**
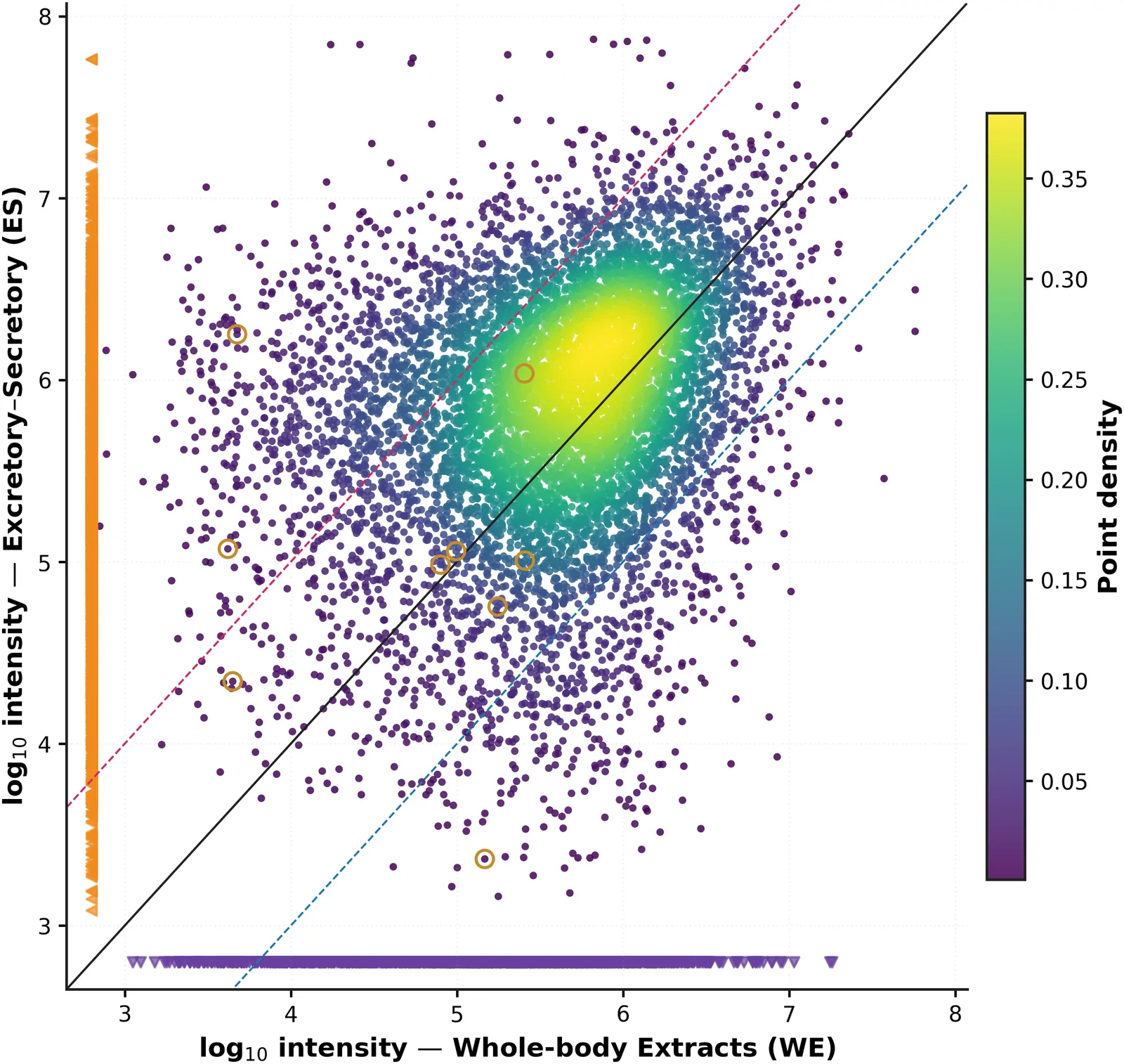
Pairwise intensity comparison of co-quantified proteins between WE and ES products of *H. ligurriens* larvae. Pairwise scatter plot of log_10_ intensities for the same co-quantified proteins (n = 7,896), colored by local point density. The solid diagonal indicates y = x, and dashed lines mark ± 2-fold boundaries. ES-exclusive proteins (n = 3,081) and WE-exclusive proteins (n = 1,548) are plotted along the left and bottom margins, respectively. Co-quantified AMP and immune-effector candidates from Table 1 (n = 9 of 34 Category 1 entries) are highlighted with gold-bordered hollow markers.

Complementary pairwise log_10_-intensity scatter analysis of co-quantified proteins demonstrated a strong overall correlation between WE and ES fractions, with most proteins distributed along the y = x identity line (Fig 3). The highest point-density region was centered at log10 intensity values of approximately 5–6 in both fractions, indicating that the majority of co-quantified proteins were detected within a comparable abundance range. Distinct fraction-specific protein partitioning was also observed, with 3,081 proteins detected exclusively in ES products and 1,548 proteins uniquely identified in WE, represented along the marginal axes. In addition, nine co-quantified putative AMP and immune-effector candidates from Table 1 were detected within the shared protein distribution. Together, these findings demonstrate both shared and fraction-specific abundance patterns within the *H. ligurriens* larval proteome. All protein identities are putative annotations based on sequence homology to the closest annotated entries in the UniProt Calliphoridae database; species matches reflect database availability and do not indicate species origin.

**Fig 4:**
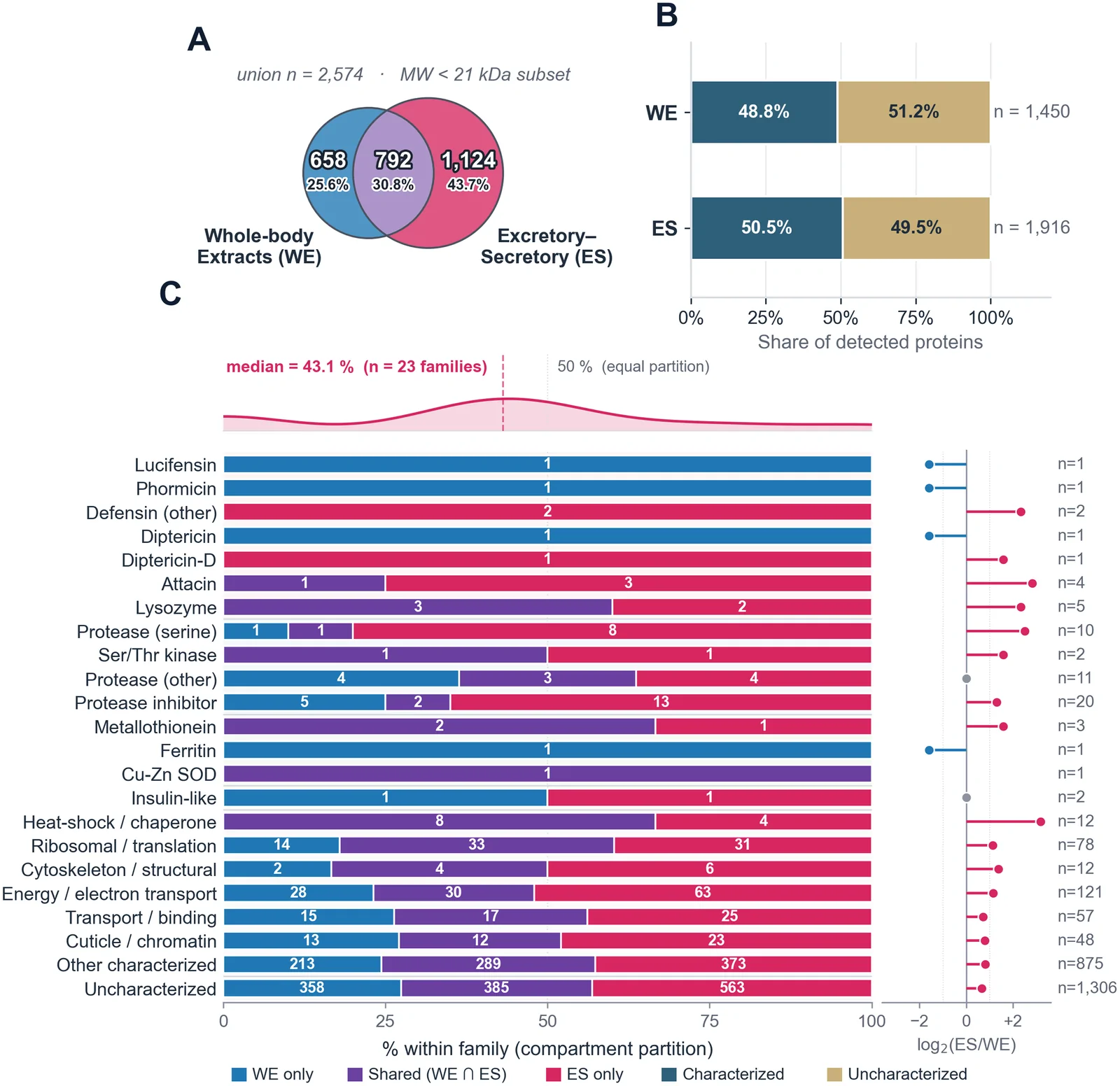
<  20 kDa subset of the *H. ligurriens* larval WE and ES products proteome-combined overview and family-resolved composition. (A) Venn diagram showing shared and uniquely identified proteins between WE and ES fractions within the < 20 kDa subset (union n = 2,574). (B) Percentage distribution of characterized and uncharacterized proteins per fraction based on homology-based annotation against the UniProt Calliphoridae database. (C) Family-resolved composition and compartment partitioning of the same subset. The main panel shows 100%-stacked horizontal bars representing per-family distribution across WE-only, shared, and ES-only proteins. The top marginal panel shows the kernel density distribution of the per-family ES-only share across the 23 non-empty families, with the cross-family median (43.1%, dashed red) and the 50% equipartition reference (dotted grey) indicated. The right panel shows family-wise log_2_ (ES/ WE) fold-change values as a lollipop plot, with reference lines indicating equal abundance (0) and ±2-fold enrichment thresholds (±1). Shared-only and empty families were excluded from the log_2_ (ES/ WE) plot. Protein families were assigned based on curated Table 1 entries and UniProt protein-name annotation.

Proteins with molecular weights below 20 kDa were selected for further analysis because antimicrobial and immune-related peptides are typically low-molecular-weight molecules. Venn diagram analysis revealed that 658 proteins (25.6%) were unique to WE, 1,124 proteins (43.7%) were detected exclusively in ES products, and 792 proteins (30.8%) were shared between the two fractions (Fig 4A). Within this subset, 1,450 proteins were identified in WE (15.4% of all proteins identified in WE), including 707 characterized proteins (48.8%) and 743 uncharacterized proteins (51.2%). In comparison, 1,916 proteins were detected in ES products (17.5% of all proteins identified in ES), comprising 968 characterized proteins (50.5%) and 948 uncharacterized proteins (49.5%) (Fig 4B). Annotation against the UniProt Calliphoridae database identified multiple bioactive proteins with molecular weights below 20 kDa in both WE and ES products, including antimicrobial peptides, immune-related proteins, and proteins annotated by sequence homology to growth factor- and cytokine-related domains. These annotations are reported based on sequence similarity and do not represent evidence of functional equivalence to mammalian growth factors or cytokines. Proteins showing compartment-specific enrichment between WE and ES products are presented in Table 1.

Family-resolved analysis of the < 20 kDa protein subset revealed distinct compartment-specific partitioning patterns between WE and ES products (Fig 4C). This subset served as the basis for the AMP and immune-effector candidate list presented in Table 1. Protein families were broadly classified into AMP/immune-effector, proteolytic, antioxidant/metal-binding, signaling, other characterized, and uncharacterized categories. Within the AMP/immune-effector category, antimicrobial peptide families included defensin, diptericin, and attacin. Defensin-related proteins were further resolved into lucifensin, phormicin, and other defensin subgroups. Several AMP- and immune-related families exhibited marked ES enrichment. Putative serine proteases showed the strongest ES-biased distribution, with eight ES-exclusive and one WE-exclusive proteins (log_2_ ES/WE ≈ +2.2). Protease inhibitors were also enriched in ES products, comprising 13 ES-exclusive and five WE-exclusive proteins (log_2_ ES/WE ≈ +1.2). In addition, attacin- and defensin-related proteins were detected exclusively in ES products, with three and two ES-only entries, respectively. In contrast, lysozyme proteins were predominantly shared between fractions, with three of five entries detected in both WE and ES. Uncharacterized proteins and residual functional categories also exhibited an ES-enriched distribution, consistent with the overall proteome-level pattern. A complete list of all putatively identified proteins, including protein names, annotations, and sample sources, is provided in S1 Table.

### Fatty acid composition of larval extracts

**Fig 5:**
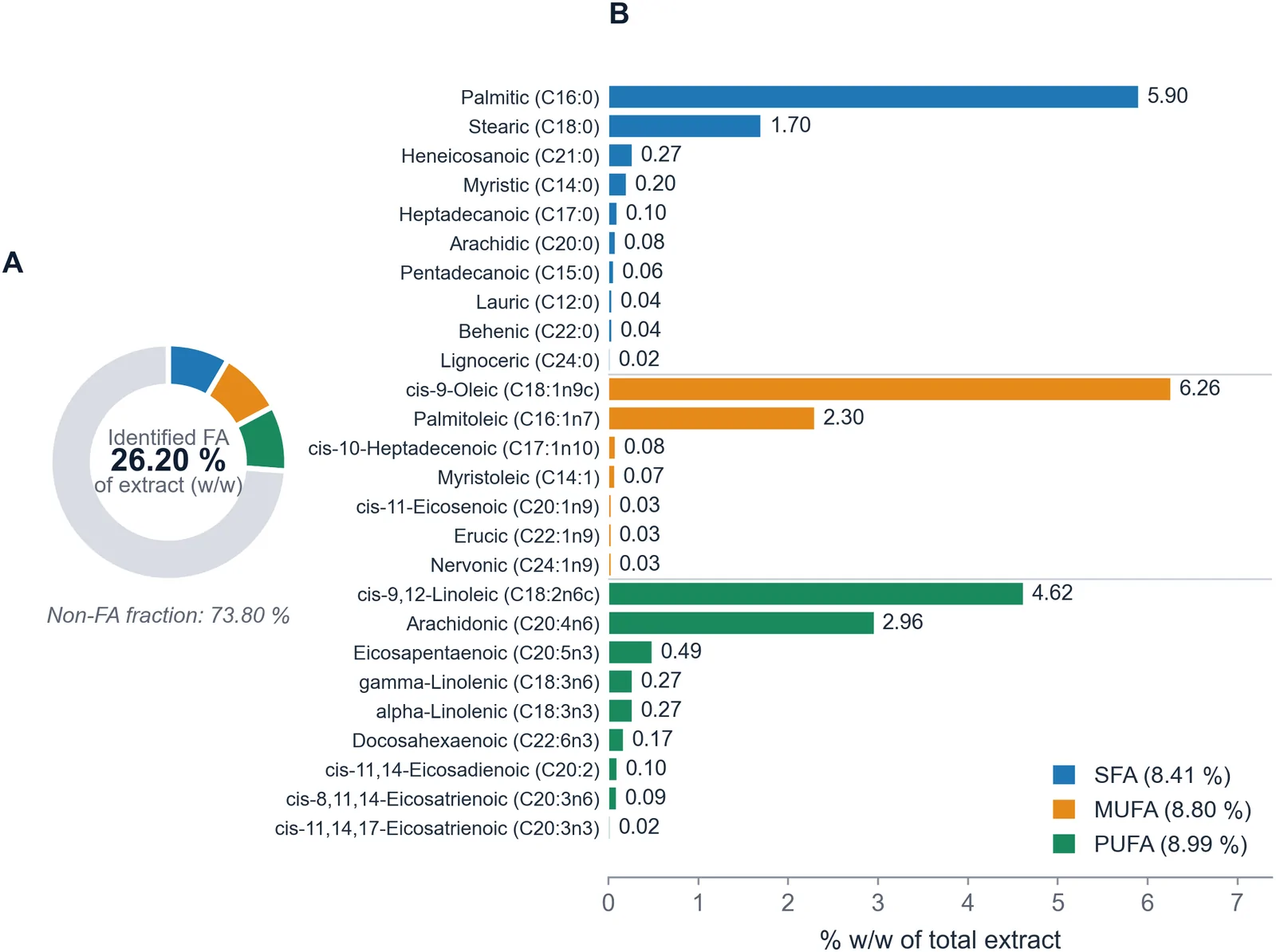
Fatty acid composition of *H. ligurriens* larval WE based on GC-MS analysis, expressed as percentage (% w/w). (A) Pie chart showing the distribution of fatty acid types, including saturated fatty acids (SFAs), monounsaturated fatty acids (MUFAs), and polyunsaturated fatty acids (PUFAs), relative to the non-fatty acid fraction. (B) Relative abundance of the 26 identified fatty acids grouped by type (SFAs, MUFAs, and PUFAs).

Fatty acid composition of the WE was determined by GC-MS analysis. As shown in Fig 5A, fatty acids accounted for 26.20% (w/w) of the extract, whereas the remaining 73.80% (w/w) comprised non-fatty acid components. The identified fatty acids consisted of 8.41% (w/w) saturated fatty acids (SFAs) and 17.79% (w/w) unsaturated fatty acids, including 8.80% (w/w) monounsaturated fatty acids (MUFAs) and 8.99% (w/w) polyunsaturated fatty acids (PUFAs). A total of 26 fatty acids were identified and classified into SFA, MUFA, and PUFA groups (Fig 5B). The predominant components were *cis*-9-oleic acid (C18:1n9c), palmitic acid (C16:0), and *cis*-9,12-linoleic acid (C18:2n6c), representing the most abundant MUFA, SFA, and PUFA, respectively, with relative abundances of 6.26%, 5.90%, and 4.62% (w/w) of the extract.

### Analysis of the antimicrobial activities of larval extracts

The antimicrobial activity of *H. ligurriens* larval extracts was evaluated using the agar well diffusion assay and a resazurin-based broth microdilution assay. Both WE and ES products produced inhibition zones against *B. subtilis* (Gram-positive bacteria), measuring 10.77 ± 0.75 mm and 16.18 ± 0.61 mm, respectively. In contrast, antibacterial activity against *P. aeruginosa* (Gram-negative bacteria) was observed only with WE (13.40 ± 3.44 mm), whereas no inhibitory activity was detected against the tested yeast or filamentous fungi under the experimental conditions. These findings indicate a selective antibacterial profile of the larval extracts (Table 2). Further evaluation by broth microdilution demonstrated that the MIC values against both *B. subtilis* and *P. aeruginosa* exceeded 400 μg/ml. Consistent with this result, resazurin remained reduced at the highest tested concentration, indicating continued bacterial growth. No bactericidal activity was detected against either bacterial species, as complete bacterial elimination was not achieved at any tested concentration (MBC > 400 μg/ml).

**Table 2:** Antimicrobial activity of WE and ES products from *H. ligurriens* larvae evaluated by agar well diffusion assay. Inhibition zones are expressed as mean ± SD (mm) from triplicate experiments.

Microorganisms	Inhibition zone (mean ± SD; mm)				
	WE	ES	Positive control	Negative control	
				Water	5% DMSO
**Gram-positive bacteria**					
*Bacillus subtilis*	10.77 ± 0.75	16.18 ± 0.61	35.09 ± 1.53	0.00 ± 0.00	0.00 ± 0.00
*Staphylococcus epidermidis*	0.00 ± 0.00	0.00 ± 0.00	26.41 ± 1.00	0.00 ± 0.00	0.00 ± 0.00
*Staphylococcus aureus*	0.00 ± 0.00	0.00 ± 0.00	30.29 ± 2.14	0.00 ± 0.00	0.00 ± 0.00
**Gram-negative bacteria**					
*Pseudomonas aeruginosa*	13.40 ± 3.44	0.00 ± 0.00	12.90 ± 1.22	0.00 ± 0.00	0.00 ± 0.00
*Escherichia coli*	0.00 ± 0.00	0.00 ± 0.00	28.f41 ± 2.15	0.00 ± 0.00	0.00 ± 0.00
*Proteus vulgaris*	0.00 ± 0.00	0.00 ± 0.00	38.70 ± 3.81	0.00 ± 0.00	0.00 ± 0.00
**Yeast**					
*Candida albicans*	0.00 ± 0.00	0.00 ± 0.00	37.23 ± 2.01	0.00 ± 0.00	0.00 ± 0.00
**Filamentous fungi**	**% Inhibition**				
	**WE**	**ES**	**Positive control**	**Negative control**	
				**Water**	**5% DMSO**
*Aspergillus flavus*	0	0	100	0	0
*Penicillium* spp.	0	0	100	0	0

**Positive control:** For all bacteria tested, tetracycline (30 μg) was used, except for *P. vulgaris* (ciprofloxacin, 5 μg). For yeast, *C. albicans* was tested with fluconazole (25 μg). For filamentous fungi, *A. flavus* and *Penicillium* spp. were tested with cycloheximide (0.5 mg). Positive controls were selected according to standard susceptibility testing guidelines for each microorganism.

**WE:** whole-body extracts (198 µg/100 µl); **ES:** excretory-secretory products (175 µg/100 µl)

Values of 0.00 indicate no detectable inhibition zone under the assay conditions.

### Effect of larval extracts on cell viability in HaCaT cells

**Fig 6:**
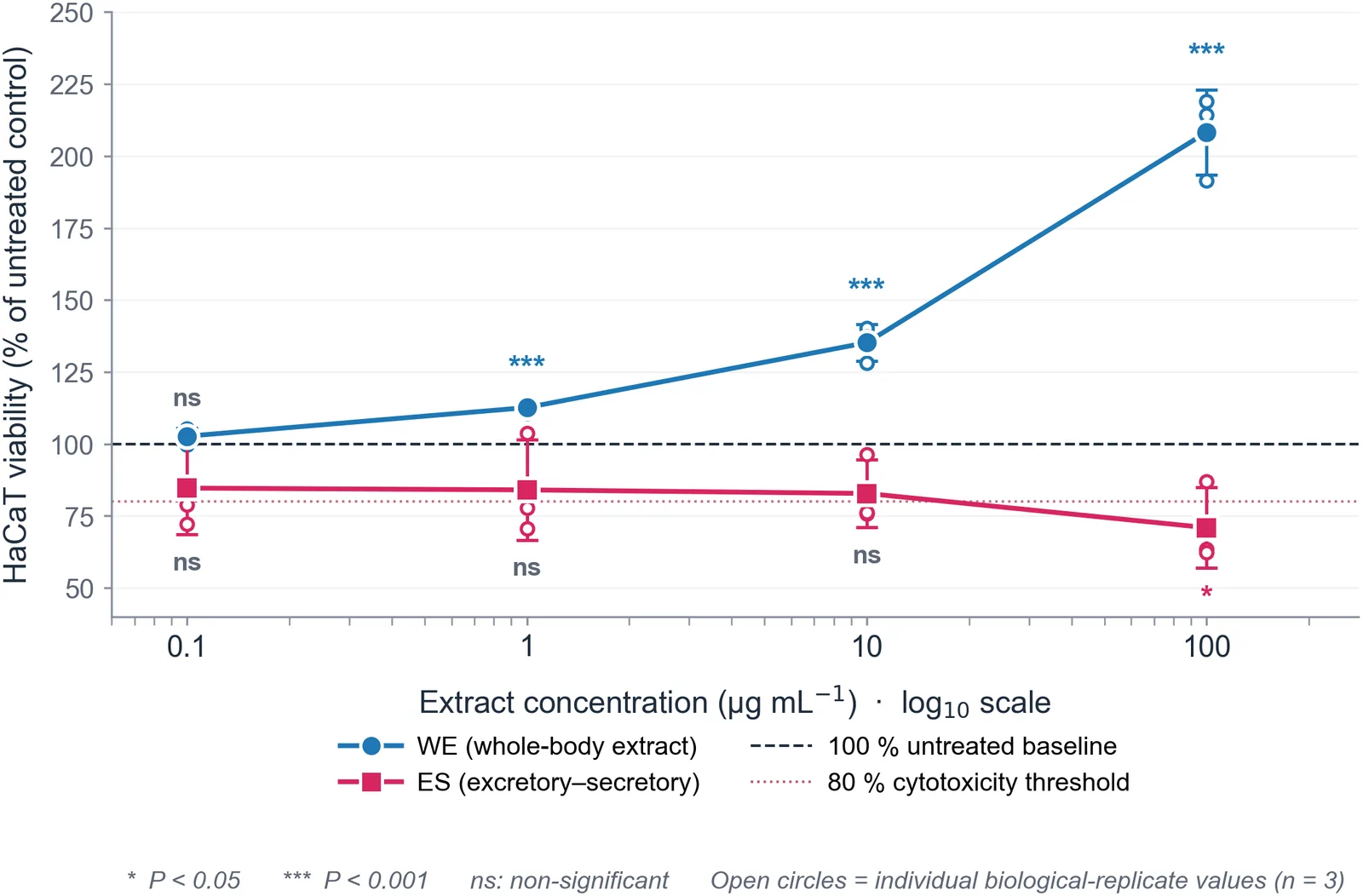
Effects of WE and ES products from *H. ligurriens* larvae on MTT-based viability of HaCaT keratinocytes. HaCaT cells were treated with varying concentrations (0.1, 1, 10, and 100 µg/ml) of WE and ES products for 24 h, and cell viability was determined using the MTT assay (n = 3 biological replicates). Data are presented as mean ± SD for WE (blue) and ES (magenta) over a log_10_ concentration axis (0.1-100 µg/ml), with individual replicate values overlaid as open circles. The 100% untreated baseline and 80% cytotoxicity threshold are indicated. Statistical significance relative to the untreated control was determined using a two-tailed Student’s t-test (*P < 0.05, ***P < 0.001, ns = non-significant). Values exceeding 100% indicate an increase in MTT signal intensity relative to the untreated control.

The effects of larval extracts on HaCaT cell viability were evaluated using concentrations of 0.1, 1, 10, and 100 μg/ml following 24 h of exposure. As shown in Fig 6, WE treatment resulted in MTT-based viability values exceeding 100% of the untreated control at all tested concentrations and displayed a concentration-dependent increase, reaching 208.27 ± 14.71% at 100 μg/ml (mean ± SD, n = 3). In contrast, ES products exhibited a concentration-dependent decrease in viability, with values ranging from 84.65 ± 16.10% at 0.1 μg/ml to 70.91 ± 13.94% at 100 μg/ml. Statistical analysis revealed that WE significantly increased MTT signal intensity at concentrations of 1, 10, and 100 μg/ml compared with the untreated control (*P* = 0.0002, 0.0006, and 0.0002, respectively). In contrast, ES products produced a significant reduction in viability only at the highest tested concentration (100 μg/ml; *P* = 0.0224). Viability remained above 80% at ES concentrations up to 10 μg/ml but decreased below this level at 100 μg/ml.

## Discussion

This study provides a comprehensive proteomic characterization and fatty acid profile analysis of WE and ES products of *H. ligurriens* larvae, a medically and forensically important blow fly species. Although the ecological and forensic relevance of *H. ligurriens* has been documented [23,24], molecular information describing its larval biochemical composition has remained limited. The present findings therefore expand the currently available molecular resources for Calliphoridae beyond better-studied species such as *L. sericata* and *L. cuprina* [5,11,14,16].

Proteomic profiling revealed marked molecular complexity in both WE and ES products, with ES containing a greater number of unique proteins than WE. Approximately four-fifths of proteins identified in each fraction were annotated as characterized, whereas the remaining proportion was classified as uncharacterized or hypothetical. The similar proportion of uncharacterized proteins between fractions suggests that this pattern is more likely attributable to incomplete database annotation in this non-model species than to extraction-related bias. The large shared core proteome, together with the substantial number of unique proteins in each fraction, supports the view that larval molecules are differentially distributed across intracellular and secreted compartments. This pattern is consistent with functional compartmentalization reported in other dipteran larvae, in which secreted fractions are enriched in environmentally interactive molecules, whereas whole-body extracts reflect broader tissue-associated physiological processes [35].

The ES fraction appeared to represent a multifunctional extracellular proteome enriched in molecules associated with antimicrobial defense, proteolysis, and stress regulation. Among the low-molecular-weight proteins, homology-based annotation identified defensin-like peptides, diptericin-like proteins, attacin-like proteins, and several lysozyme isoforms. These peptide classes are well-recognized components of dipteran innate immunity [5,12,13], and their detection in ES products is consistent with the observed antibacterial activity against *B. subtilis*. ES products also contained proteolytic enzymes, including chymotrypsin and several serine proteases. In blow fly larvae, these enzymes have been associated with extracellular matrix degradation and promote fibrinolysis [4,44]. In addition, recombinant chymotrypsin has been reported to interfere with bacterial adhesion and disrupt biofilm formation [45]. Notably, chymotrypsin was detected exclusively in ES products, in agreement with previous reports in larval secretions of *L. cuprina* and *L. sericata* [4,46]. Because proteolytic enzymes in medicinal maggot secretions have also been implicated in debridement and tissue remodeling [4], their presence in *H. ligurriens* may suggest potential relevance to wound-associated biological activity, although direct functional validation remains necessary.

Several stress-related and antioxidant-associated proteins were also detected in the ES fraction, including Cu/Zn superoxide dismutase. These molecules may contribute to oxidative stress modulation in the highly microbe-rich larval environment and may also be relevant to wound-associated contexts, in which redox balance influences tissue repair. Similar interpretations have been proposed for larval extracts of other calliphorid species, including *C. albiceps*, which showed antioxidant and wound-related bioactivity *in vitro* [8]. Previous studies have reported wound-associated biological activities in larval secretions of *L. sericata* [47,48]. In the present study, proteins with annotations related to growth factor- and cytokine-associated signaling were also detected; however, these annotations should be interpreted cautiously because homology-based identification does not establish functional equivalence to mammalian wound-healing factors. Direct biochemical and cellular validation will be necessary to confirm whether these proteins contribute to tissue-relevant activity.

In contrast to ES products, WE represented a broader composite fraction derived from multiple larval tissues, including the fat body, digestive tract, epithelium, salivary glands, hemocytes, and other organs [5,11,14,15,28,29]. Accordingly, WE contained a wider range of proteins associated with metabolism, detoxification, antioxidant defense, structural support, and immune function. This broader biochemical profile suggests that WE capture both constitutive intracellular immune components and inducible stress-response molecules involved in systemic physiological regulation. Several molecules detected exclusively in WE, including lucifensin, ferritin, and phormicin, further support this interpretation. Lucifensin has previously been identified in multiple tissues and body compartments of *L. sericata* and *L. cuprina*, including gut, fat body, salivary glands, hemolymph, and WE, where it has been recognized as a major defensin peptide with antimicrobial activity [11,14,16]. Similarly, phormicin from *Musca domestica* exhibits strong antibacterial activity, including membrane-disruptive and antibiofilm effects [49,50]. Ferritin, which has also been characterized in *M. domestica*, is known to participate in iron homeostasis, antioxidant defense, and immune protection [51]. In most insects, including *Drosophila melanogaster*, ferritin is predominantly found in the hemolymph [52]. The detection of lucifensin and phormicin exclusively in WE may reflect tissue storage, differential secretion, or limitations in peptide recovery from ES products under the extraction and analytical conditions employed in this study. Together, these findings suggest that WE may contain a mixture of direct antimicrobial molecules and broader physiological effectors that indirectly shape microbial survival.

In this study, methanol-soluble lipid profiling of WE from *H. ligurriens* larvae revealed a diverse mixture of saturated and unsaturated fatty acids, with oleic acid, palmitic acid, linoleic acid, and palmitoleic acid among the major constituents. Comparable fatty acid profiles have been reported from WE of *Hermetia illucens* and *L. sericata* larvae [30,38], suggesting that these lipid classes may represent common components of dipteran larval lipid extracts. In addition to the major constituents, several other fatty acids, including myristic, stearic, pentadecanoic, and arachidic acids, were also detected. This indicates substantial lipid diversity within the larval extracts, consistent with previous reports [39]. Several fatty acids identified in WE have previously been associated with antimicrobial activity. Lauric, palmitoleic, oleic, linoleic, α-linolenic, eicosenoic, palmitic, and stearic acid have been reported to inhibit microbial growth primarily through disruption of membrane integrity and permeability, resulting in leakage of intracellular contents and impairment of essential cellular functions [30–32,53–55]. In addition to their antimicrobial properties, some of these fatty acids have been associated with antioxidant and anti-inflammatory activities [39,56]. The presence of multiple bioactive fatty acids therefore suggests that lipid-associated molecules may contribute to the biological activities observed in WE. The broader antibacterial activity observed in WE compared with ES products may be partly attributable to the presence of both lipid-associated molecules and putative antimicrobial peptides within the same fraction. The concurrent detection of bioactive fatty acids together with putative antimicrobial peptides supports the possibility that the antibacterial activity of WE results from the combined effects of multiple molecular classes rather than a single active constituent. However, the individual antimicrobial contributions of the detected fatty acids were not directly evaluated in the present study. Furthermore, because lipid profiling was restricted to methanol-soluble extracts, the resulting profile likely represents only a subset of larval lipid-associated constituents and should not be interpreted as a comprehensive characterization of the *H. ligurriens* larval lipidome.

The functional assays support the molecular findings. WE inhibited both *B. subtilis* and *P. aeruginosa*, whereas ES products inhibited only *B. subtilis*, and no antifungal activity was observed under the tested conditions. Similar selective antibacterial patterns have been reported in other calliphorid larvae [35,41,57,58]. The broader activity of WE, particularly against *P. aeruginosa*, may reflect the combined contribution of peptide-associated and lipid-associated molecules. Although ES products contained a greater diversity of putative antimicrobial peptides in the proteomic analysis, they did not exhibit detectable antibacterial activity against Gram-negative bacteria under the tested conditions. A similar pattern was reported for ES products of *C. megacephala*, which showed antibacterial activity against Gram-positive bacteria but limited activity against Gram-negative species despite the presence of multiple putative antimicrobial peptides [35]. This discrepancy may reflect differences in peptide abundance, accessibility to bacterial targets, or the limited permeability of Gram-negative outer membranes to peptide-based antimicrobials. The MIC and MBC values of >400 μg/ml indicate only moderate antibacterial potency under the present assay conditions. Previous studies reported antibacterial activity of *L. sericata* larval ES products against both Gram-positive and Gram-negative bacteria, including *Staphylococcus epidermidis*, *S. aureus*, and *P. aeruginosa*, with MIC values ranging from 100 to 400 μg/ml depending on the bacterial strain and assay tested [59]. In the present study, *H. ligurriens* larval products also produced measurable inhibition zones against both Gram-positive (*B. subtilis*) and Gram-negative (*P. aeruginosa*) bacteria in the agar diffusion assay using less than 200 μg of WE and ES products. The apparent discrepancy between inhibition-zone formation and relatively high MIC values may reflect differences in assay format, diffusion behavior, and extract complexity. These findings suggest that the crude extracts contain bioactive molecules that are present at relatively low abundance, exhibit limited potency in crude form, or occur in combinations that are not optimally resolved in bulk preparations. In addition, agar diffusion assays provide only a qualitative or semi-quantitative estimate of activity and may underestimate poorly diffusing or hydrophobic compounds [41]. The antimicrobial evaluation was also limited to a small number of test microorganisms. Future studies should therefore include a broader range of pathogens and more comprehensive assays, such as biofilm inhibition, time-kill kinetics, and synergistic interaction analyses, to better characterize antimicrobial activity. Further fractionation, purification, and targeted testing of candidate peptides and lipid fractions will be required to define antibacterial potency more precisely and to identify the principal active components.

Beyond antimicrobial activity, the larval extracts showed favorable cytocompatibility in human HaCaT keratinocytes. WE exhibited no cytotoxicity across the tested concentration range and significantly increased MTT-based metabolic activity after 24 h exposure. Because the MTT assay primarily reflects cellular metabolic activity, these findings should not be interpreted as direct evidence of cell proliferation or wound-healing efficacy. ES products likewise maintained high viability at lower concentrations, although viability declined at 100 μg/ml, indicating mild dose-dependent cytotoxicity at the highest concentration tested. Similar non-cytotoxic and wound-associated biological effects have been reported for larval extracts of several calliphorid species, including *C. megacephala*, *C. albiceps*, *L. sericata*, and *Sarconesiopsis magellanica* [8,27,35,58]. Collectively, these findings suggest that *H. ligurriens* larval extracts are compatible with further investigation in skin- and wound-related biomedical applications. However, the present study was limited to MTT-based cytocompatibility assessment and did not evaluate cellular injury, migration, or wound-repair responses. Additional *in vitro* and *in vivo* studies, including LDH release, apoptosis-related markers, scratch wound assays, and cytokine profiling, will therefore be required to determine their therapeutic relevance.

Overall, the findings support the hypothesis that WE and ES products represent distinct molecular compartments with different bioactive repertoires. ES products were characterized by a greater proportion of exclusive proteins and contained numerous defense-, proteolysis-, and immune-associated molecules, including diptericin-like proteins, attacin-like proteins, lysozymes, and chymotrypsin. In contrast, WE represented a broader tissue-derived molecular reservoir containing diverse metabolic, antioxidant, immune-related, and lipid-associated constituents. Notably, several putative antimicrobial peptides, including lucifensin and phormicin, were detected exclusively in WE, indicating that antimicrobial effectors are distributed across both fractions rather than being confined to ES products. These molecular differences were reflected in the functional assays, in which WE exhibited broader antibacterial activity than ES products. Collectively, these findings support compartment-specific molecular organization between externally interactive larval secretions and systemic larval tissues. Although the current annotations are based primarily on homology and the functional assays were performed using crude preparations, this study provides an important molecular resource for a medically and forensically important yet understudied tropical calliphorid species. Because proteomic analyses were performed on pooled samples with technical replicates, the observed proteomic differences should be considered exploratory and warrant validation using independent biological replicates and quantitative proteomic approaches. In addition, because larvae were maintained under standard laboratory conditions without microbial challenge, the identified proteomic profile likely reflects constitutive defense-associated molecules. Future studies should investigate the effects of immune stimulation, including microbial exposure or experimental infection, on AMP expression and antimicrobial activity, while also prioritizing peptide and lipid purification, targeted functional validation, and wound-healing assays to define the molecular basis and translational potential of these larval bioactivities.

## Conclusions

This study provides the first integrative proteomic and lipidomic characterization of *H. ligurriens* larvae, revealing compartment-specific distributions of bioactive molecules between WE and ES products. Proteomic analysis identified diverse defense-, immune-, and proteolysis-associated molecules, including putative antimicrobial peptides, whereas lipidomic profiling of WE revealed multiple fatty acids with reported antimicrobial properties. Notably, antimicrobial effectors were distributed across both fractions, with several putative antimicrobial peptides, including lucifensin and phormicin, detected exclusively in WE. Functionally, WE exhibited broader antibacterial activity and favorable *in vitro* cytocompatibility, whereas ES products showed more selective antibacterial effects. Together, these findings expand the molecular resources available for tropical calliphorid flies and provide a foundation for future studies aimed at functional validation, bioactive compound discovery, and biomedical applications.

## Supporting information

S1 Table Proteins identified by LC-MS/MS in WE and ES products of *H. ligurriens* larvae. The table presents protein identifiers and annotations, number of proteins, peptide counts, sequence coverage, molecular weight, sequence length, Q-value, identification score, protein intensity in the WE and ES samples, and MS/MS count. An intensity value of zero indicates that the protein was not detected in the corresponding sample. WE, whole-body extracts; ES, excretory-secretory products; LC-MS/MS, liquid chromatography-tandem mass spectrometry.
(XLSX)

S1 Code Python source code and accompanying input data for statistical analysis and visualization. The ZIP file contains Python scripts and input data used to analyze and visualize the proteomic, fatty-acid composition, and cytocompatibility datasets presented in this study. The accompanying README file provides instructions, software requirements, and methodological notes for reproducing the analyses.
(ZIP)
